# Biochar dispersion in a tropical soil and its effects on native soil organic carbon

**DOI:** 10.1371/journal.pone.0300387

**Published:** 2024-04-18

**Authors:** Alfred Obia, Jing Lyu, Jan Mulder, Vegard Martinsen, Gerard Cornelissen, Andreas Botnen Smebye, Andrew R. Zimmerman

**Affiliations:** 1 Department of Agronomy, Faculty of Agriculture and Environment, Gulu University, Gulu, Uganda; 2 Department of Geological Sciences, University of Florida, Gainesville, Florida, United States of America; 3 Faculty of Environmental Sciences and Natural Resource Management (MINA), Norwegian University of Life Sciences (NMBU), Aas, Norway; 4 Norwegian Geotechnical Institute (NGI), Oslo, Norway; Government College University Faisalabad, Pakistan, PAKISTAN

## Abstract

Although biochar application to soils has been found to increase soil quality and crop yield, the biochar dispersion extent and its impacts on native soil organic carbon (SOC) has received relatively little attention. Here, the vertical and lateral migration of fine, intermediate and coarse-sized biochar (<0.5, 0.5–1 and 1–5 mm, respectively), applied at low and high doses (1.5–2 and 3–4% w/w, respectively), was tracked using stable isotope methods, along with its impact on native SOC stocks. Biochar was homogeneously mixed into the surface layer (0–7 cm depth) of a loamy sandy Acrisol in Zambia. After 4.5 y, 38–75% of the biochar carbon (BC) was lost from the applied layer and 4–25% was detected in lower soil layers (7–30 cm). Estimating BC mineralization to be no more than 8%, 25–60% was likely transported laterally out of the experimental plots. This conclusion was supported by observations of BC in the control plot and in soils up to 2 m outside of the experimental plots. These processes were likely progressive as recovery of BC in similar plots 1 year after application was greater in both surface and lower soil layers than after 4.5 y. Fine and intermediate-sized BC displayed the greatest downward migration (25.3 and 17.9%, respectively), particularly when applied at lower doses, suggesting its movement through soil inter-particle spaces. At higher dosages, fine and intermediate-sized particles may have clogged pore, so coarse biochar displayed the greatest downward migration when biochar was applied at higher doses. In the BC treatment plot soil profiles, native SOC stocks were reduced by 2.8 to 24.5% (18.4% on average), i.e. positive priming. However, some evidence suggested that the soils may switch to negative priming over time. The dispersion of biochar in soil should be considered when evaluating biochar’s agronomic benefits and environmental effects.

## Introduction

Biochar is a carbon-rich soil amendment produced by the pyrolysis of biomass that has many potential applications for carbon sequestration, soil fertility improvement, and remediation of contaminated soils. However, the longevity of positive agronomic and climate mitigating effects of biochar are central questions in the biochar research community due to the general lack of long duration field studies. Physical presence of biochar in crop fields will certainly be a determining factor in the long-term effects of biochar on soil properties and soil productivity. However, biochar may be mobile in the soil environment, migrating beneath the root zone or beyond the crop field [1, 2], thus reducing or eliminating the expected positive effects of biochar, such as increases in total soil carbon content, cation exchange capacity, and water holding capacity [3, 4].

The potential biochar dispersion in soil is multidirectional. Obia et al. [2] reported that lateral transport of biochar through surface ‘floating‘, as well as by erosion, accounted for most of the biochar carbon (BC) dispersion in a one-year experiment in the coarse-textured soils of Zambia. This loss accounted for up to 45% of the BC applied to the upper 7 cm of the soil, whereas downward transport accounted for 10–20%. High rates of lateral transport (erosion) of biochar or similar carbonaceous materials has also been reported in a number of other studies [1, 5–7]. For example, Rumpel et al. [8] found that eroded soils were 2.3 times enriched in BC, on average, compared to native soil organic carbon (SOC), in a steep tropical setting. In a controlled rainfall experiment conducted on a gentle (1%) slope [6], 7–55% of the BC added to the soil through surface grass fires was subject to horizontal transport.

Significant downward transport of BC has also been noted in several studies. For example, about 50% of a biochar produced from rice residues may have moved below the upper 30 cm sandy soil layer in which it was applied over 4 y [9]. In low density peatland soils previously used to dispose of household burn residues, BC migration rates were calculated as 0.6–1.2 cm y^−1^, with 21–69% of BC migrating from the surface down to a maximum of 1.4 m over 95 y [10]. In contrast, Major et al. [11] found that only a small portion (0.5%) of the BC applied to a savanna Oxisol in Colombia moved below the upper 10 cm application depth over 2 y. Similarly, only < 10% of the BC applied to the upper 3 cm of boreal forest soils was recovered below that depth interval after 2 y [12].

The dispersion of biochar in soil away from the point of application may be influenced by a number of factors ranging from inherent physiochemical soil and biochar attributes, to climate and field management practices. Soils with high clay contents may promote incorporation of biochar particles into soil aggregates [13], which may reduce the downward and lateral transport of biochar. Physical attributes of biochar, such as ease of disintegration into smaller particles, which varies with feedstock and field aging, may increase its tendency to migrate [14]. The preferential erosion of BC, versus SOC, has also been reported [6, 8, 15]. Finally, climate can certainly be a controlling factor, as regions prone to high wind or high intensity rainfall events will likely have greater biochar erosion [7].

Given the stable nature of BC, total soil carbon is expected to increase upon incorporation of BC into soil. In addition, BC may increase native SOC because of its positive effect on biomass production by enhancing nutrient and water retention [16]. But biochar can also increase SOC by decreasing its rate of mineralization (negative priming) through such mechanisms as sorptive protection, exoenzyme inhibition, or other organo-mineral interactions [17]. But application of biochar to soil can also increase SOC mineralization (positive priming) through co-metabolization or by stimulating soil microbial activity. A number of factors have been found to influence the magnitude and direction of biochar priming in soils, including land use [18], soil carbon content [19], soil organic matter composition [20], soil pH [21], biochar feedstock [17, 22] and biochar pyrolysis temperature [23]. Soils with low pH and low TOC concentration that are common in tropical areas may be more vulnerable to positive priming effects, i.e. native SOC loss upon biochar application, at least in the short term [19, 21]. A number of studies reported the transient nature of the priming effect of biochar, with a switch from positive to negative priming with time [17, 20, 24]. The longer-term negative priming after biochar application may be responsible for increased native SOC stocks in a subtropical ferralsol after 8 y [25] and in Amazon *terra preta* [26] produced by biochar additions 100’s of years ago.

Despite, this understanding that biochar can both migrate and influences mineralization of native SOC, there are few studies that have both monitored biochar movement in the soil profile over longer time periods (>1 y) and none that simultaneously examined its effect on native SOC priming. Based on the above findings, it is hypothesized that both lateral and downward transport of BC will continually occur and will be greater with biochar of smaller particle size. It is also hypothesized that by 4.5 y after biochar application to a low carbon tropical soil, biochar will have a negative priming effect, and increases in native SOC stocks will be observed both in upper soil layers to which biochar has been added, as well as in lower layers into which biochar has migrated. To test these hypotheses, a loamy sand agricultural soil at Mkushi, Zambia was amended with corn cob biochar of different particle size fractions and at different dosages. To test for vertical BC transport, samples were collected from different soil depths within the amended plots. To test for lateral BC transport, samples were also collected adjacent to the experiment plots. Both BC recovery and changes to native SOC stocks in the soil profile due to biochar addition were quantified using a ^13^C isotopic mixing model. Novel aspects of the present study include long-term tracking of various particle sizes and dosages of BC, and changes in SOC in the soil depth profile along with lateral migration.

## Materials and methods

### Field experiment

The field experiment was established in April 2013 in Mkushi, Zambia (S13 44.839, E29 05.972) on flat terrain with a natural vegetation of open miombo typical of southern Africa forest. The land was used for maize cropping over the past 10–15 years. The regional climate is humid sub-tropical with dry winters and the average annual precipitation and temperature at Mkushi is 1220 mm and 20.4°C, respectively. The experiment was conducted on private land and oral approval was obtained from the land owner.

The soil at the site is classified as an Acrisol according to FAO’s World Reference Base classification [27]. These soils are widespread globally and dominate central and western Zambia. While the productivity of these soils has been shown to increase with biochar addition [28, 29], as a well-drained sandy loam low density soil, they might be expected to see losses of surface-applied biochar to deeper layers.

**Fig 1:**
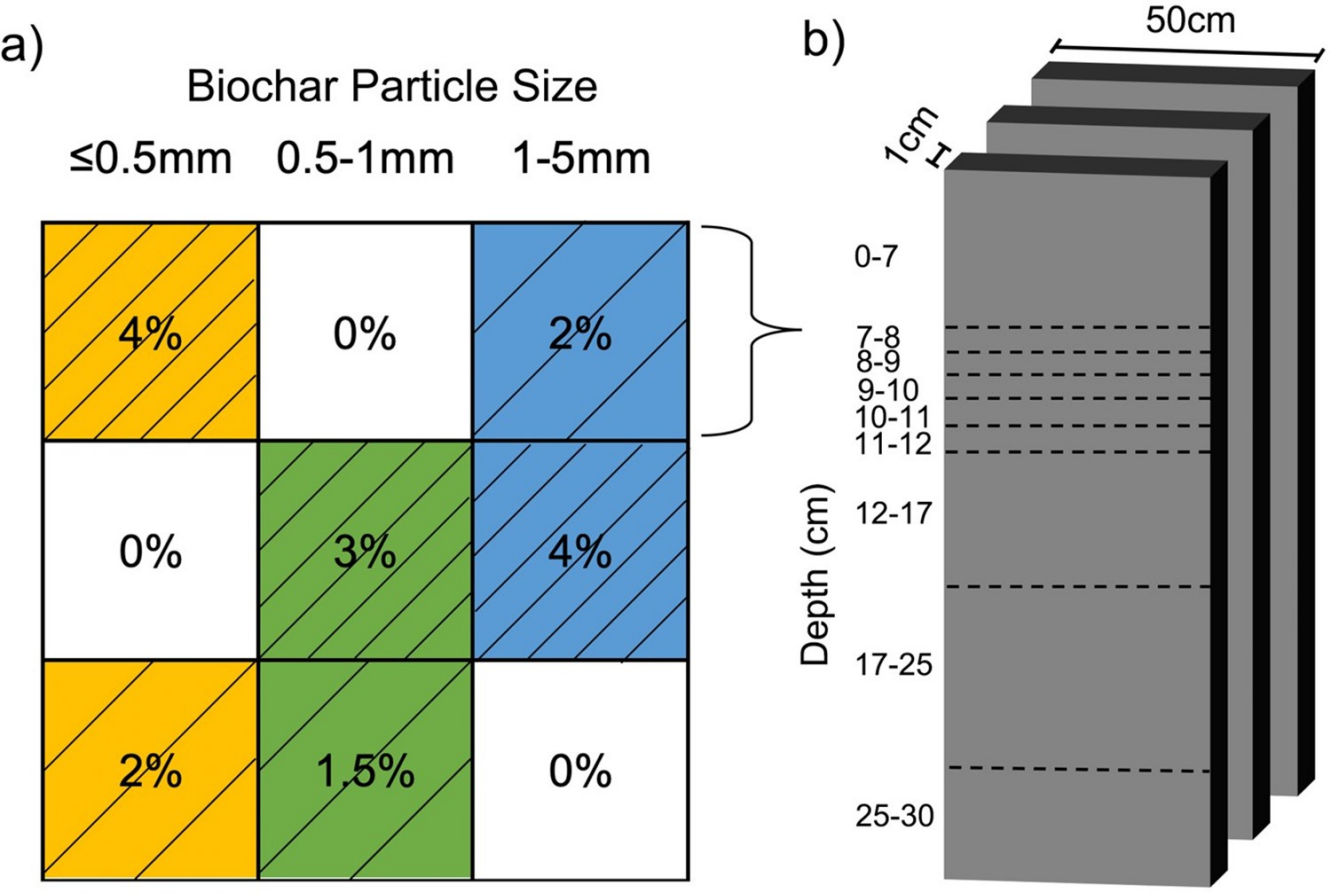
Schematic of experimental design. (A) Layout of 50 x 50 cm plots. Numbers in boxes indicate biochar dose by weight percent and yellow, green, and blue colors represent fine, medium and coarse sized biochar, respectively. (B) 1 x 50 cm (length x width) samples collected from each plot over depth intervals indicated.

The experiment was arranged as three blocks of nine 50 cm x 50 cm plots (Fig 1A). Each experimental block consisted of three biochar particle sizes and three biochar application rates (n = 3 for each treatment). The first block was sampled after 1 y and reported on previously [30]. The second block was sampled after 4.5 y for this study and the third was, unfortunately, disturbed and unusable.

Each plot was enclosed with hard plastic sheets inserted vertically to a depth of 10 cm into the soil and extending 10 cm above the soil. Prior to biochar application, all plots were hand-loosened to a depth of 30 cm, including the Bt horizon, using a Chaka hoe, a common agricultural process in Zambia. Treatments consisted of three biochar particle sizes (< 0.5, 0.5–1 and 1–5 mm, referred to as fine, intermediate and coarse biochar, respectively), prepared by crushing and sieving. These particle size cutoffs were chosen based on prior finding that each represented about one-third of the biochar produced (unpublished data). Using the bulk densities of the soil and each biochar reported previously [3], the fine and coarse size biochar was applied at a rate of 0% (reference; BC0), 2% (BC2) and 4% (BC4), by weight, to a 7 cm soil depth, amounting to 0, 0.437 and 0.873 kg per plot, respectively. These additions correspond to about 4 and 8 metric t ha^-1^ and are within the range of common regional biochar application rates [29, 31]. Due to shortage of material, the intermediate size biochar was applied at slightly lower rates of 0%, 1.5% (BC1.5) and 3% (BC3), corresponding to 0, 0.333 and 0.666 kg biochar per plot, respectively.

As a ubiquitous waste biomass in Zambia and the greater southern African region, as well as its lack of competing uses, the biochar was prepared from dry corncob, i.e. cobs with grains removed, in a retort kiln at a temperature of approximately 400 to 500°C with a residence time of 24 hrs. Details of other production conditions can be found in Sparrevik et al. [32]. During the biochar application, the uppermost 7 cm of soil from each plot, including the reference plot, was removed before loosening the soil further to 30 cm depth. The required amount of each biochar was homogenized with soil in a bucket by hand hoe and spade, facilitated by the dryness of the soil, and returned to the plot. The plots were planted with maize and fertilized with ‘Compound D’ (N, P_2_O_5_, K_2_O, 10:20:10), applied at a rate of 200 kg ha^-1^ y^-1^ with a top dressing of urea at 140 kg ha^-1^ during each growing season (approximately November to March) over the 4.5 y.

### Soil sampling

Samples were collected by excavating three 50 x 1 cm slices from each of nine plots in the block (Fig 1B) in September 2017, 4 y and 5 months after biochar application. Three replicate samples were collected from nine soil depth intervals; 0–7 cm (biochar application depth), and 7–8, 8–9, 9–10, 10–1 1, 11–12, 12–17, 17–25 and 25–30 cm. Further, to assess the lateral transport of biochar, triplicate 50 x 1 cm soil samples were also collected from the 0–7 cm depth interval of soils outside the experimental blocks at distances of 0.5, 1, 2 and 5 m to the north and south. However, it should be acknowledged that all three experimental blocks may have contributed to the biochar recovered outside the experimental blocks. Each depth interval sampled was homogenized by hand mixing, dried, ground and stored for later analyses. The field-moist soil samples were dried at 40°C for 3 days before homogenization by crushing and passing through a 2 mm sieve. Subsamples were milled for isotopic and TOC analysis.

### Laboratory analyses and data handling

Basic soil and biochar properties such as texture, bulk density, pH, cation exchange capacity (CEC), soil carbon, nitrogen and hydrogen content, and base cations were analyzed using standard procedures, as provided in Obia et al. [2, 30], and are reported along with biochar loss on ignition and soluble constituents (Table 1). The δ^13^C signatures of the original soil and biochar and collected sample mixtures were measured using a ThermoFinnigan DeltaPlus XL isotope ratio mass spectrometer with a GasBench II universal online gas preparation device at the University of Florida, Florida, U.S. Values were calibrated relative to a CO_2_ reference gas and all isotopic ratios are expressed in standard delta notation as δ^13^CO_2_ in units of per mil (‰) relative to the Vienna Pee Dee Belemnite (VPDB) standard. The analytical precision based on replication of standards was ±0.1‰.

**Table 1:** Soil and biochar properties.

Properties	Soil	Maize cob biochar^1^	
		≤0.5 mm	Bulk
Sand (%)	75.1	-	-
Silt (%)	15.9	-	-
Clay (%)	9.0	-	-
Bulk density (g cm^-3^)	1.26^2^	-	-
Total organic C (%)	0.74	44.8	53.8
Total nitrogen (%)	0.01	0.79	0.65
Total hydrogen (%)	-	2.09	2.36
pH	5.8	9.0	8.8
Loss on ignition (%)	-	52.1	-
Soluble constituents (%)	-	2.6	2.4
Surface area^3^ (m^2^ g^-1^)	-	10.5	-
CEC (cmol_c_ kg^-1^)	1.7	-	22.2
K^+^ (cmol_c_ kg^-1^)	0.3	-	16.5
Ca^2+^ (cmol_c_ kg^-1^)	1.1	-	4.3
Mg^2+^ (cmol_c_ kg^-1^)	0.3	-	1.2
δ^13^C (‰)	-19.5	-12.3	-12.3

^1^Maize cob biochar of particle size 0.5–1 mm was not characterized because it was fully used in the field. Mass balance calculation of 0.5–1 mm maize cob biochar was based on C content of the ≤ 0.5 mm fraction. The δ^13^C of the soil presented here is the average of the bulk soil at 7–30 cm depth sampled in September 2017.

^2^Bulk density of the top soil (0–7 cm) from the reference plot.

^3^BET surface area analyzed via N_2_ sorptometry.

The difference in δ^13^C isotope signature of the SOC and the BC allowed the calculation of the proportion of C from each source using a two-component isotope mixing ratio equation:

$$BC\;Fraction\;(f)=\frac{{\delta^{13}C}_{mixture}-{\delta^{13}C}_{ref\;soil}}{{\delta^{13}C}_{biochar}-{\delta^{13}C}_{ref\;soil}}$$ (1)

where δ^13^C mixture is the δ^13^C of total organic carbon (TOC) in a given soil sample at the time of collection. The BC (g) in a given sample was calculated as:

BiocharC(BC)=TOCxf (2)

The total native SOC or BC stock for each depth interval was calculated as:

$$C\;stock\;(g)=Plot\;area\;({cm}^{2})*Depth\;range\;(cm)*bulk\;density\;(g\;{cm}^{-3})*\frac{C\%}{100}$$ (3)

using the final soil bulk density measured at each depth interval (S5 Table in S1 File). The non-BC carbon stock, here also called native SOC, from which biochar priming effect was assessed, was obtained by subtracting BC stock from the TOC stock.

### Statistical analysis

The data was analyzed using one-way ANOVA in R software (R Team, 2016) where treatments (consisting of control, i.e. reference plot, biochar particle size fraction and dose, e.g. fine BC at 2%, intermediate BC at 4%, etc.) were considered as categories. The dependent variables were δ^13^C signature, TOC concentration, TOC stock, BC and non-BC (or native SOC) stock and BC recovery. Pairwise t tests were conducted in R software for above dependent variables to identify which treatments differed from the reference plot. Significant difference was accepted at a p < 0.05 level.

## Results

All data collected is provided in S5 Table in S1 File of the supplementary information section, whereas the means of triplicated treatments are referred to in the following. While significant differences were found between native and pyrogenic carbon concentrations in the each soil horizon, there were high levels of variability in replicates, somewhat reducing the power of detection of differences. The relative standard deviation of BC content was 23.6% in the upper soil application layer, on average, but 80%, on average, overall. The relative standard deviation of non-BC native content was much lower, at 11.3%, on average.

### Soil profile Δ^13^C and C content variation

The initial δ^13^C signatures of the soil and biochar were -19.5 and -12.3‰, respectively (Table 1). Thus, after 4.5 y of field emplacement, irrespective of biochar particle size and dose, the δ^13^C isotope signature of the amended soils was significantly greater than that of the reference plot within the biochar application depth of 0–7 cm (S2a Fig in S1 File). This effect was more pronounced for plots amended with coarse biochar (low and high dosage p < 0.001), followed by the fine biochar (low dosage p = 0.009, high dosage p < 0.001), and less so for the intermediate particle size biochar (low dosage p = 0.014, high dosage p = 0.003). The δ^13^C within the application depth of the reference plot also increased during the 4.5 y experiment, from -19.5 to -17.6‰, suggesting some lateral movement of biochar into the reference plot. Despite all biochar size treatments showing elevated δ^13^C isotope signatures, only the high dosage fine biochar (p = 0.023) and low and high dosage coarse biochar (p = 0.012) treatments had significantly greater C contents within the application depth compared to the reference plot (S1 Table in S1 File).

Below the biochar application depth interval, there was no evidence of biochar presence in the reference plots (i.e., no δ^13^C signatures were significantly different from -19.5‰) after 4.5 y. In the treatment plots, soils immediately below the biochar application depth, i.e. 7–9 cm, and low doses of 2% fine biochar resulted in the most pronounced increases in δ^13^C isotope signature and C content compared to other treatments. Significant increases in δ^13^C isotope were also observed deeper in the soil profiles (down to 25 cm), but mainly for treatments with low dosages of fine and intermediate biochar size fractions and high dosage of coarse biochar. Below this depth, in the 25–30 cm depth interval, the δ^13^C signatures of the treated soils were not significantly different from that of the reference soil.

The TOC stock throughout the soil profile was also not consistently affected by biochar addition (S1 Table in S1 File). While the fine biochar high dose treatment plots had significantly greater TOC stock than the reference plot (by 18%), in all other cases, total C stocks were not significantly different after 4.5 y. This is likely due to BC loss via horizontal migration as discussed below.

### Biochar carbon recovery variation with soil depth

**Fig 2:**
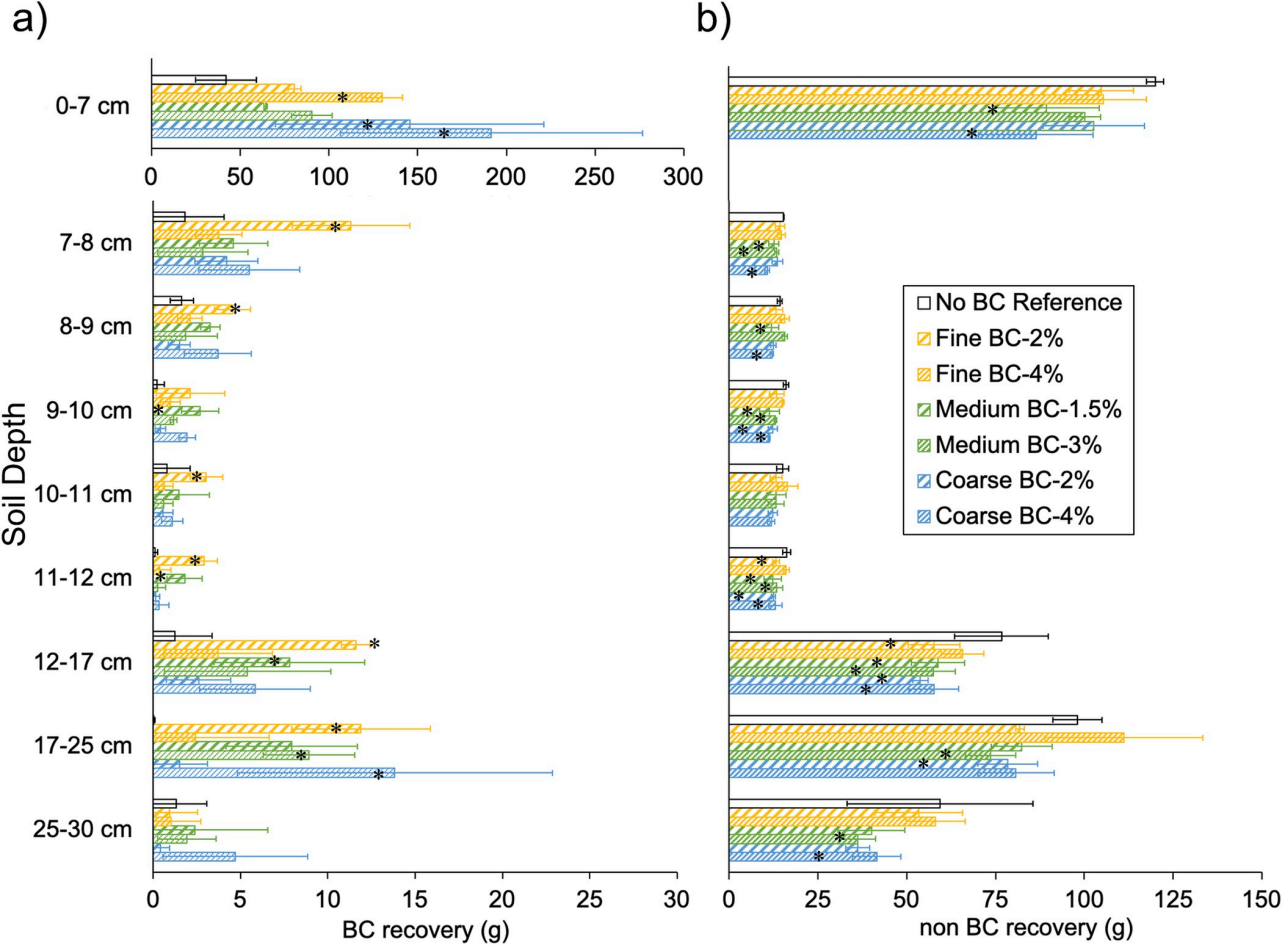
Soil profile of (A) biochar carbon (BC) recovery, and (B) native soil organic carbon (non-BC) measured after 4.5 y field emplacement with biochar applied in the 0–7 cm depth interval. Bars represent depth averages with standard error across three replicate samples. Asterisks indicate values that are significantly different from that of the reference plot (no biochar added). Yellow, green, and blue colors indicate coarse (1–5 mm), intermediate (0.5–1 mm) and fine (< 0.5 mm) particle sizes, respectively and striping pattern differentiates low (1.5–2%) and high (3–4%) biochar dosages for each particle size class.

The amount of BC calculated to be in the soil within the 0–7 cm biochar application depth after 4.5 y varied with both biochar particle size and dosage (Fig 2A and S2 Table in S1 File). Although all biochar treatment plots had average BC contents greater than those of the reference plots within the application depth, because of high variation among the replicates, the only plots with significantly greater amounts were the 4% dosage fine biochar (p = 0.048), and both the 2 and 4% dosage coarse biochar treatments (p = 0.047 and 0.013, respectively). The recovery rate of BC in the application layer ranged from 25.2%, in the high dosage intermediate-sized treatment plot to 61.9% in the low dosage coarse-sized treatment plot.

Almost all the subsurface (7–30 cm) soil layers of biochar treatment plots had average BC amounts greater than those of corresponding reference plot layers, but again, due to high variation, relatively few layers had significantly greater amounts (p = 0.004, 0.029 and 0.015 for 2% dosage fine biochar, 1.5% dosage intermediate biochar, and 4% dosage coarse biochar, respectively). This implies significant downward BC migration. Below the biochar application depth, most of the depth intervals above 25 cm of treatments amended with lower biochar dosages (1.5–2%) of fine and intermediate particle sizes contained significantly greater amounts of BC than in the reference plots. Plots with greater biochar dosages (3–4%) less commonly had significantly greater amounts of BC in comparison to the reference plot. In contrast, the amount of BC in low dosage coarse treatments recovered in any layers below the application depth did not differ from those in the reference plots, indicating low overall downward migration for this treatment. The total recovery rates of BC in soil layers below the application depth in the amended plots ranged from 25.3% in the low dosage fine treatment plot to 3.8% in the high dosage fine biochar treatment plot.

Total profile BC recovery from the amended plots after 4.5 y ranged from 32 to 67% and generally decreased from coarse to fine to intermediate-sized treatments (S2 Table in S1 File). Greater proportions of BC were recovered from low dose versus higher dose biochar plots, irrespective of particle sizes. This, along with detection of BC, both in the reference plot, where no biochar was applied, and in samples collected from soils outside the plot area, strongly suggests that there was significant lateral transport of BC. About 49.1 g of BC was detected in the reference soil profile after 4.5 y, the great majority of which was in the upper 7 cm (S2 Table in S1 File). After 4.5 y, two of the six samples collected within 2 m of the treatment blocks had detectable BC (11.0 and 7.0 g BC, calculated for an area of 0.5 x 0.5 m), while none of the four samples collected at a distance 3 and 5 m distance had detectable BC (S5 Table in S1 File).

### Effect of biochar on native SOC

The mean native SOC (i.e. non-BC carbon) in all treatment plot soil horizons was almost always less than or not significantly different from that of the non-amended reference plots (Fig 2B and S3 Table in S1 File). However, only low dose intermediate-sized (p = 0.046) and high dose coarse biochar (p = 0.039) treatment plots had significantly less SOC (25%, on average) within the 0–7 cm application layer compared to the reference plot. Below the biochar application depth, intermediate and coarse biochar treatments had significantly reduced amounts of native SOC stock (p < 0.05) within most sampled depth intervals, irrespective of biochar dosage. In contrast, fine biochar treatments largely did not significantly affect the amount of native SOC in any depth interval (except in two cases). With the exception of the fine biochar high dosage treatment, all biochar treatments had significantly reduced native SOC stocks in the whole-profile (by 15.3 to 24.5%) and in the portion of the profile below the application depth (by 16.3 to 25.6%).

## Discussion

### Biochar mobility in soil

The rates of BC recovery within the upper 7 cm application depth ranged 25–62% (S2 Table in S1 File), implying that a significant fraction of the applied BC (38–75%) had been lost. Such high losses of BC from soil have also been found in an irrigated silt loam Fluvisol in northeast. China (40% of BC over 5 y) where 1.1 to 3.3 wt % biochar was applied to the upper 20 cm of the soil and tilled each year [33]. Between 20 and 53% of BC was lost from a sandy tropical Oxisol over 2 y [11] in Columbia where rainfall was twice that of the Zambia site. In the current study, some BC losses may have occurred via mineralization. Annual BC losses due to mineralization, according to a meta-analysis of field and laboratory incubation studies lasting more than 1 y, averaged 1.8% y^-1^ [34]. This can be used to calculate an estimate of potential BC mineralization loss of about 7.8% over the 4.5 y of our study. The rate is probably much less as mineralization may be inhibited by the long dry season in Zambia, and because mineralization rates have been shown to decrease asymptotically over time [35]. Assuming this maximum of 7.8% BC mineralization, 33.3 to 68.4% of BC can be calculated to have been lost to vertical or lateral migration.

### Vertical mobility

One fate of the BC lost from the applied layer was migration to lower layers of the soil profile where 3.8 to 25.3% of the applied BC was found. As significant BC was detected in the 17–25 depth interval, movement from 7 cm to 17 cm would imply a minimal BC migration rate of 2.2 cm y^-1^, much greater than the 0.6–1.2 cm y^-1^ rate of Leifield et al. [10] working in drained peatland soils where rainfall was similar to the of the Zambia site. From ^14^C dating of charcoal fragments found at 1 m soil depth, Carcaillet [36] calculated a downward transport rate of 2 mm yr^-1^ over multiple centuries in the northwestern Alps. The higher rate of vertical movement of BC observed in the current study may be due to the sandy nature of the soil and the relatively high and seasonal rainfall that occurs in the region. In addition to the larger pore spaces of sand-sized particles, the greater mobility of biochar in sandy soil could be due to the lesser amounts of clays and polyvalent cations, which minimize the incorporation of biochar into stable aggregates [37]. This is in line with the much higher vertical movement of biochar observed in a sandy Arensol, compared to a Cambisol and a Ferralsol [38]. Another factor that may have increased vertical migration was the hand hoe-loosening of soil to a depth of 30 cm prior to biochar application.

Losses of BC from the upper soil layer to which biochar was applied ranged from 38.1% in the low dose coarse biochar treatments to 74.8% in the high dose intermediate-sized biochar treatments. So clearly, the mobility of BC in soil was also found to be affected by biochar particle size and dosage. At low doses (1.5 or 2%), fine and intermediate-sized BC displayed greatest downward migration, likely because these smaller particles were able to fit within soil inter-particle spaces and move downward with percolating water. Previous studies that considered biochar particle size found smaller particles to be more readily transported downward through porous media [39, 40]. Some fine biochar particles may have attained colloid size (< 53 μm) during crushing and sieving, which further increases its downward mobility [41]. Another potential mechanism of downward transport of BC is in dissolved form via leaching and adsorption to soil minerals at greater soil depths. Finer biochar particles lose a greater fraction of their carbon in dissolved form via leaching [42]. However, this was unlikely to be a major mechanism as only about 0.2 to 7.4% of BC has been found to be leachable [43].

Much less downward BC migration was detected at the higher biochar dosages, particularly for fine and intermediate-sized biochar treatments, possibly indicating that clogging of soil pores may have occurred. In contrast, greater downward migration of coarse versus finer BC occurred in higher dose treatments. According to Obia et al. [3], coarse biochar applied at the same site was more effective than fine biochar in creating macro-pores (>30 μm diameter), i.e. packing voids. These voids, at higher dosages, may have acted as passages for later downward BC migration, since BC particles disintegrate over time [14]. In addition, high doses of coarse biochar may reduce soil-biochar interaction, as was found for a Vertisol [44], which would inhibit the formation of stable aggregates, thus increasing BC migration.

**Fig 3:**
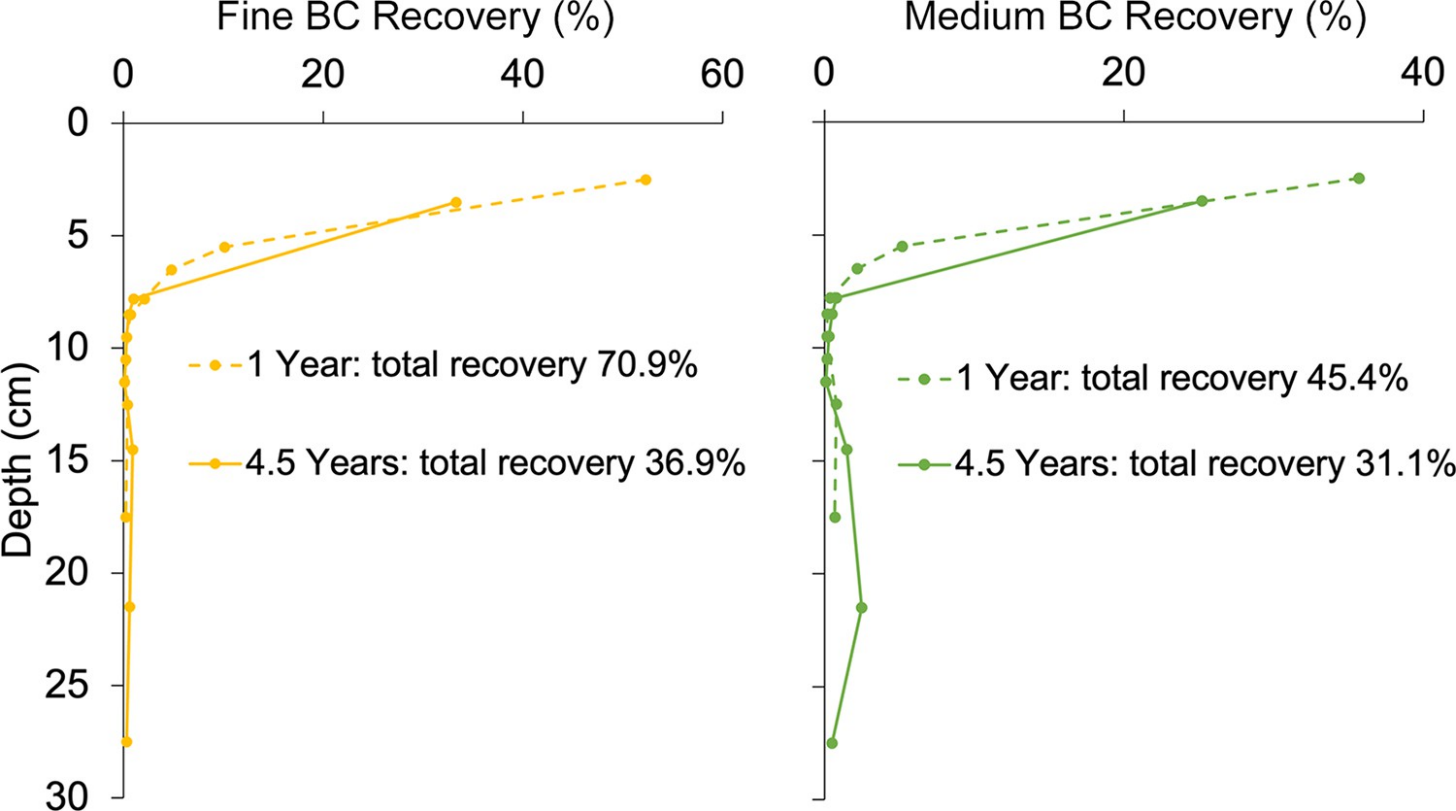
Recovery of maize cob biochar carbon (BC) from soil profiles with treatments fine (< 0.5 mm, left panel) and intermediate (0.5–1 mm, right panel) particle size biochar 1 y (dashed line) and 4.5 year (solid line) after application. One year data is from Obia et al. (2017a) where BC application depth was 0–5 cm instead of 0–7 cm in the current study.

Comparison of BC recovery rates after one year of field emplacement, as reported in Obia et al., [2], with those reported here after 4.5 y (Fig 3 and S4 Table in S1 File), suggest that BC downward migration was an ongoing process. Of the fine and intermediate-sized biochar that was recovered from high dose treatment plots (coarse and low dose treatments were not a part of the 1 y study), 5.3 and 5.2% of the BC was found below the application depth after 1 y, respectively, whereas 10.2 and 20.3% was found in these lower layers after 4.5 y. The BC had also moved over time to progressively deeper soil layers, with about 2 to 3 times more BC recovered from soil layers deeper than 10 cm after 4.5 y compared to 1 y. To our knowledge, this is the first study to document progressive downward transport of BC through the soil column.

### Lateral mobility

Of the BC lost from the application layer in the current study, about 8% may have mineralized and ~4–25% was found in underlying soil, down to 30 cm, leaving a larger fraction of at least 25–60% unaccounted for. While a small portion may have migrated below the tested soil column, the majority of this fraction was likely lost laterally through erosion as was further supported by the finding of BC in the reference plot and outside the experimental block. Similar high BC erosional losses of surface-applied BC was reported by Major et al. [20–53%, 11] where rainfall was twice that of the Zambia site and Rumpel et al. [7–55%, 6] where rainfall was similar but the terrain was steeply sloped. Thus, lateral erosion may be the main BC dispersion avenue in many cases. However, loss rates can be quite variable. Recoveries of grass BC applied to high latitude cold and dry climate Cryosols and Regosols ranged from 11 to 39% after 2 y [12]. The coarse texture (loamy sand) of our study site may have facilitated erosion of BC due to limited soil aggregation and little incorporation of BC into aggregates as shown by Obia et al. [3]. Similarly, Schiedung et al. [45] found that BC dispersion from point of application was greatest in coarse versus finer-textured soils. Similar to downward migration, lateral transport loss was also shown to be a progressive process as less total BC was recovered from the plot profiles after 4.5 year versus after 1 year (37.1 versus 71.4% for fine biochar and 31.6 versus 45.5% for intermediate size biochar).

The proportions of fine and coarse BC recovered in the studied soil profile (0–30 cm) were greater than that of the intermediate-sized BC after both 1 and 4.5 y. This lower recovery of intermediate-sized BC (~54 and 32% for low and high dose treatments, respectively, within the studied profile; S2 Table in S1 File) may be explained by its disproportionally greater lateral erosion. This might be due to the greater ability of fine BC to migrate downward in the soil profile, thus avoiding erosion, and the greater weight of coarse biochar, which could have inhibited its transport by wind. Water transport, however, might be unaffected by size as biochar has a dry bulk density much less than water, allowing it to float [46].

In each particle size category, there was much lower total recovery of BC from high dose compared to low dose biochar treatment plots, implying greater lateral transport from the former. This is in line with hypothesis that greater downward migration of coarse BC, when applied at high dosages, was due to reduced soil-biochar interaction which limited the formation of stable aggregates that also inhibit erosion. There has been very little study of the influence of biochar particle size and dosage on both vertical and lateral BC migration, so the results and mechanisms proposed here require much further study.

### Native soil carbon stock and biochar priming effect

According to a recent meta-analysis of studies that examined biochar priming effect on SOC [34], biochar addition had a small negative priming effect, on average, when applied to sandy soils (clay content < 10%) such as present at our study site. However, while TOC stock generally increased in the surface soil layer where biochar was applied, a decrease in the native SOC stock was also generally observed. This occurred in all of the treatment plots within the application layer, in many of the soil layers below the application depth, and in the total soil profiles (18.4% SOC loss, on average), relative to the reference plot. This suggests that positive priming occurred due to biochar addition over the 4.5 y of the study. Positive priming can be caused by stimulation of microbial activities, either through the addition of labile substrates or nutrients, or because of improvements to soil conditions such as pH and moisture levels [47]. Given the acidic nature of the Mkushi soil (pH 5.8), the high pH of the biochar used (pH 8.3) would likely have had a stimulatory effect. This also may have increased native SOC solubility [48], thus causing SOC desorption from mineral surfaces and subsequent mineralization. The positive priming found in this study are in line with results of Cross and Sohi [19] and Luo et al. [21] who reported greater SOC mineralization upon biochar addition to soils with low pH and low carbon content.

Another observation was much greater positive priming of native SOC in the intermediate and coarse biochar amendment plots. In the total soil profiles, intermediate and coarse biochar addition reduced SOC stocks by 22–24% while fine biochar reduced native SOC stock by only 3% and 16% for high and low doses, respectively (Fig 2B and S3 Table in S1 File). This might be explained by the timing at which priming proceeds. Several *in vitro* incubation studies reported positive priming of SOC in the early period after biochar addition to soil, which switched to negative priming afterward as the labile substrate was depleted and the biochar began to act as a sorbent of soil organic matter, protecting it from microbial exoenzyme degradation [17, 20, 23, 49]. A meta-analysis of biochar priming studies [50] found that, on average, positive priming occurred within the first 2 y of incubations, with a change to negative priming afterwards. In the open system of the Mkushi soil, the continual movement of dissolved and particulate BC to lower soil layers may have extended the period of positive priming. This may also explain lesser degree of positive priming observed in the biochar application layer, which may have already switched to negative priming. Similarly, because fine biochar was more mobile than the other size fractions, it may have already switched from positive to negative priming. Thus, less loss of native SOC was observed in the fine biochar amendment plots.

While losses of native SOC were observed, the high recalcitrance and carbon-rich nature of biochar compensated for the lost native SOC. Mean TOC (BC + non-BC) measured in each plot profile was greater or not significantly different from that of the reference plot. Further, the observations and associated mechanisms proposed above imply that the priming effect of biochar may be transient and will switch to greater degrees of negative priming with time. That is, biochar may, with time, increasingly protect native SOC from degradation, similar to what has been reported previously [e.g.,17, 25, 50]. Future studies should monitor the fate of the added BC and its effect on native SOC at even greater timescales.

## Conclusions and implications

While definitive conclusions as to variations in migration among different biochar types and the effects of biochar migration on total C stocks were limited somewhat due to high variability among replicates, it is clear that field-applied biochar is highly mobile. As hypothesized, downward transport of BC was shown to be an ongoing process during the 4.5 y experiment, though erosional loss of BC from the experimental plots was the quantitatively greatest mode of dispersion. Our hypothesis that smaller particle-sized biochar would show the greatest downward migration proved to be generally true, but this was dose-dependent.

The hypothesis that biochar application would have a negative priming effect was not supported. The migration of BC downward in the soil profile caused positive priming, presumably by stimulating the decomposition of soil organic matter, reducing native SOC stock. However, evidence suggested that this may be a transient effect and that some treatment plots had transitioned from positive to negative priming over time. In any case, it may be inadvisable to plough biochar deeply into the soil subsurface, since this may further stimulate loss of native SOC.

The significant lateral transport of the applied biochar (25–60% of BC) led to non-significant increases in total C stock in many of the experimental plots with biochar additions. The treatments of fine and intermediate-sized biochar applied at the lower dosages showed the lowest rates of erosion, possibly because of its higher soil infiltration (downward migration), suggesting that this may be the preferred mode of application to achieve greatest agricultural benefits most efficiently. However, it should be mentioned that fine biochar is more difficult to handle and can potentially pose an inhalation hazard. Other approaches to minimize erosion may be to apply biochar to leveled, terraced, or contoured plots so that biochar stays within planted areas. However, given that BC movement was detected only as far as 2 m from its application location, and loss from one location is gain for another in a larger field setting, the net lateral loss from a field may be quite small.

The mobility of BC in soil, both downward and laterally, may limit the benefits of biochar that are unrelated to C sequestration, which may be agronomic (e.g., enhanced soil quality or increased crop yields) or environmental (e.g., reduced N_2_O emissions or contaminant adsorption). The movement of biochar and, by extension, pyrogenic carbon produced during prescribed and wildfires, may have implications for ecological and human health. How biochar dispersion may or may not impact adjacent wetlands and aquatic systems should be studied. Further work should be conducted to examine how soil properties and climate may influence pyrogenic carbon migration, as well as to further examine the relationship between biochar migration and BC and native SOC degradation/preservation.

## Supporting information

S1 File Contains **S1 Fig**. Photos of biochar field application at different stages at Mkushi, Zambia. Photos taken by J. Mulder in April 2013. From A. Obia 2015 Doctorate Thesis; **S2 Fig.** Soil profiles of a) δ^13^C isotope signatures and b) total carbon contents after 4.5 y field emplacement with biochar applied in the 0–7 cm depth interval. Bars represent depth average values with standard error across three replicate samples. Asterisks indicate mean values that are significantly different from that of the reference plot (no biochar added). The vertical dashed line in the left panel indicates the δ^13^C of the original soil (-19.5‰). Yellow, green, and blue colors indicate coarse (1–5 mm), intermediate (0.5–1 mm) and fine (<0.5 mm) particle sizes, respectively and striping indicates low (1.5–2%) and high (3–4%) biochar dosages for each particle size class; **S1 Table.** Mean total organic carbon stock (g) in the soil profile 4.5 years after biochar addition; **S2 Table.** Mean quantities and recovery rates of maize cob biochar carbon (BC) recovered by depth interval in the soil profile, 4.5 years after application; **S3 Table.** Mean native soil organic carbon (non-biochar C, g) 4.5 years after biochar addition; **S4 Table.** Comparison of mean biochar carbon (BC) recovery from soil profiles, 1 and 4.5 years after biochar application; and **S5 Table.** Compilation of data used in this study.
(PDF)
