# Supplementary material for: Biochar dispersion in a tropical soil and its effects on native soil organic carbon
Source: PLoS One. 2024 Apr 18;19(4):e0300387. doi: 10.1371/journal.pone.0300387 (PMC11025871; doi:10.1371/journal.pone.0300387)
Supplement: S1 File — Contains S1 Fig. Photos of biochar field application at different stages at Mkushi, Zambia. Photos taken by J. Mulder in April 2013. From A. Obia 2015 Doctorate Thesis; S2 Fig. Soil profiles of a) δ13C isotope signatures and b) total carbon contents after 4.5 y field emplacement with biochar applied in the 0–7 cm depth interval. Bars represent depth average values with standard error across three replicate samples. Asterisks indicate mean values that are significantly different from that of the reference plot (no biochar added). The vertical dashed line in the left panel indicates the δ13C of the original soil (-19.5‰). Yellow, green, and blue colors indicate coarse (1–5 mm), intermediate (0.5–1 mm) and fine (<0.5 mm) particle sizes, respectively and striping indicates low (1.5–2%) and high (3–4%) biochar dosages for each particle size class; S1 Table. Mean total organic carbon stock (g) in the soil profile 4.5 years after biochar addition; S2 Table. Mean quantities and recovery rates of maize cob biochar carbon (BC) recovered by depth interval in the soil profile, 4.5 years after application; S3 Table. Mean native soil organic carbon (non-biochar C, g) 4.5 years after biochar addition; S4 Table. Comparison of mean biochar carbon (BC) recovery from soil profiles, 1 and 4.5 years after biochar application; and S5 Table. Compilation of data used in this study. (PDF) [file pone.0300387.s001.pdf]

Supplementary Information

Biochar dispersion in a tropical soil and effects on native soil organic carbon

Alfred Obia[1], Jing Lyu[2], Jan Mulder[3], Vegard Martinsen[3], Gerard Cornelissen[3, 4], Andreas Botnen Smebye[4], Andrew R. Zimmerman[2,*]

[1] Department of Agronomy, Faculty of Agriculture and Environment, Gulu University, Gulu, Uganda

[2] Department of Geological Sciences, University of Florida, Gainesville, Florida, USA.

[3] Faculty of Environmental Sciences and Natural Resource Management (MINA), Norwegian

[4] Department of Sustainable Geosolutions, Norwegian Geotechnical Institute (NGI), Oslo, Norway

[5] University of Life Sciences (NMBU), Aas, Norway

* Corresponding author: azimmer@ufl.edu

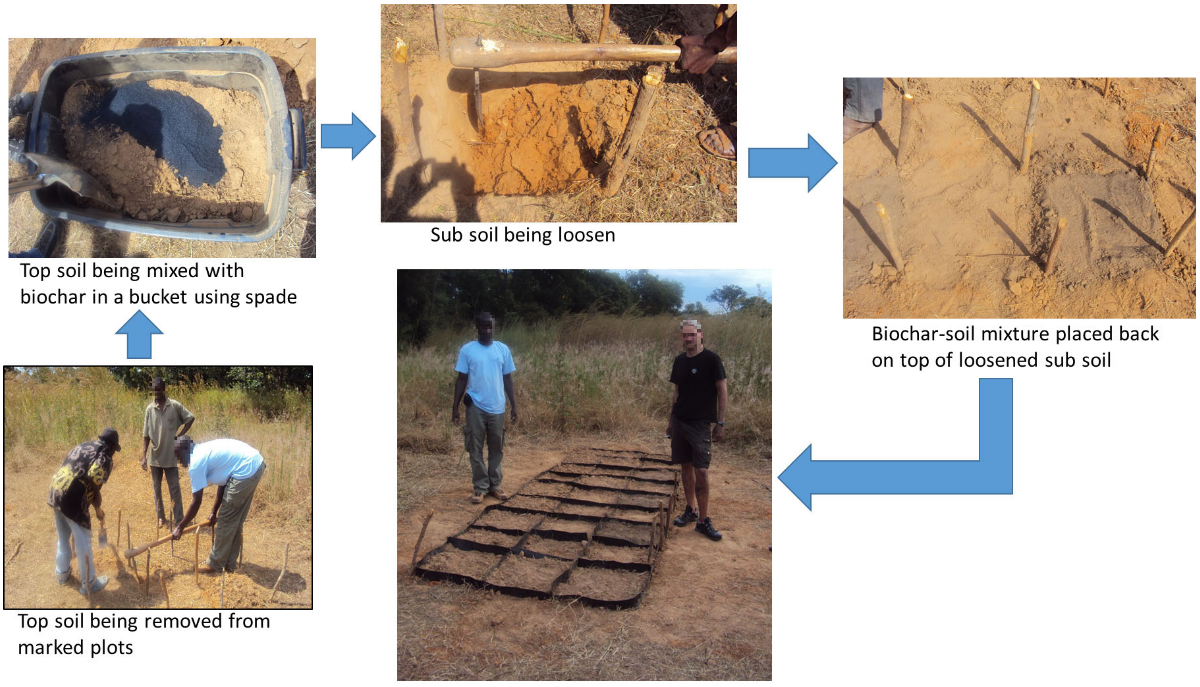

Figure S1. Photos of biochar field application at different stages at Mkushi, Zambia. Photos taken by J. Mulder in April 2013. From A. Obia 2015 Doctorate Thesis.

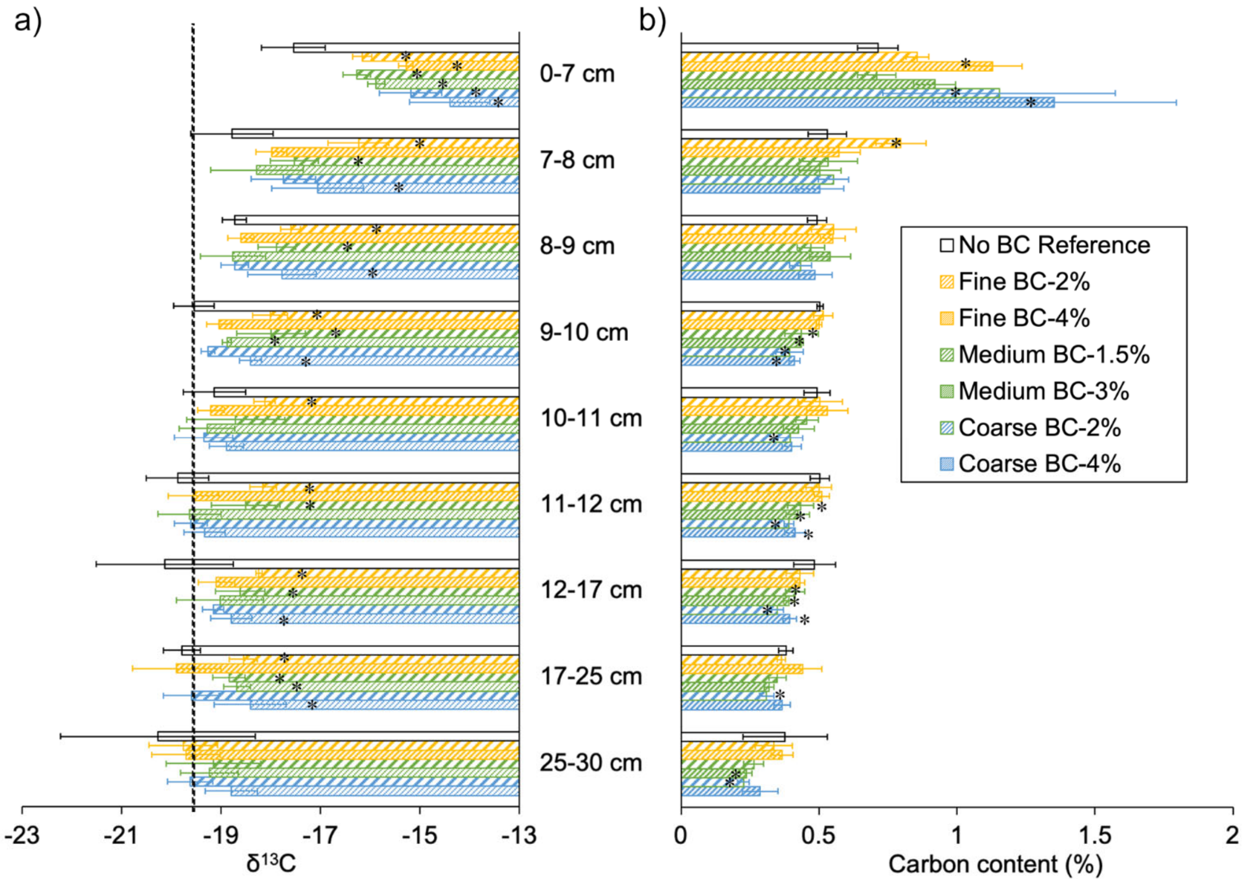

Figure S2. Soil profiles of a) $\delta^{13}C$ isotope signatures and b) total carbon contents after 4.5 y field emplacement with biochar applied in the 0–7 cm depth interval. Bars represent depth average values with standard error across three replicate samples. Asterisks indicate mean values that are significantly different from that of the reference plot (no biochar added). The vertical dashed line in the left panel indicates the $\delta^{13}C$ of the original soil (-19.5‰). Yellow, green, and blue colors indicate coarse (1-5 mm), intermediate (0.5-1 mm) and fine (<0.5 mm) particle sizes, respectively and striping indicates low (1.5 – 2%) and high (3 – 4%) biochar dosages for each particle size class.

Table S1. Mean total organic carbon stock (g) in the soil profile 4.5 years after biochar addition[1].

Soil depth	Reference	<0.5 mm BC		0.5–1 mm BC		1–5 mm BC	
(cm)	BC0	BC2	BC4	BC1.5	BC3	BC2	BC4
0 – 7	157.3	181.4	231.4*	150.3	186.8	244.2*	274.7*
7 – 8	17.1	25.7*	18.5	17.2	16.2	17.8	16.2
8 – 9	15.9	17.8	17.7	15.2	17.4	14.0	15.7
9 – 10	16.2	16.6	16.1	14.1*	14.1*	12.7*	13.2*
10 – 11	15.9	16.2	17.1	14.7	13.8	12.8	12.9
11 – 12	16.2	16.1	16.4	14.0*	13.7*	12.6*	13.3*
12 – 17	77.9	69.3	69.3	66.7*	62.9*	56.4*	63.4*
17 – 25	98.0	93.7	113.5	90.3	82.6	80.0*	94.6
25 – 30	60.7	54.3	59.1	42.5	38.2*	36.6*	46.2
Total	475.4	485.7	559.2*	424.9	445.5	487.1	550.4

[1] calculated on a 0.5 x 0.5 m plot size basis.

Asterisks indicate mean values significantly different from that of reference plot for each soil depth layer, t test at $p \leq 0.05$.

Table S2. Mean quantities and recovery rates of maize cob biochar carbon (BC) recovered by depth interval in the soil profile, 4.5 years after application.

Soil depth (cm)	Reference	<0.5 mm BC particles				0.5–1 mm BC particles				1–5 mm BC particles			
	BC0 (g)	BC2 (g[1])	% recovery	BC4 (g)	% recovery	BC1.5 (g)	% recovery	BC3 (g)	% recovery	BC2 (g)	% recovery	BC4 (g)	% recovery
0 – 7	41.9	80.6	41.2	130.0*	33.3	64.3	35.9	90.3	25.2	145.4*	61.9	191.5*	40.8
7 – 8	1.8	11.3*	5.8	3.8	1.0	4.7	2.6	2.8	0.8	4.2	1.8	5.5	1.2
8 – 9	1.7	4.6*	2.3	2.1	0.5	3.2	1.8	1.9	0.5	1.5	0.6	3.7	0.8
9 – 10	0.2	3.2	1.6	1.0	0.3	2.7*	1.2	1.2	0.3	0.4	0.2	1.9	0.4
10 – 11	0.8	3.0*	1.6	0.6	0.2	1.5	0.6	0.6	0.2	0.6	0.2	1.1	0.2
11 – 12	0.1	2.9*	1.5	0.4	0.1	1.8*	0.8	0.3	0.1	0.1	0.1	0.3	0.1
12 – 17	1.2	11.6*	5.9	3.7	0.9	7.8*	3.3	5.4	1.5	2.6	1.1	5.8	1.2
17 – 25	0.0	11.9*	6.1	2.4	0.6	7.9	3.4	8.9*	2.5	1.5	0.6	13.8*	2.9
25 – 30	1.3	1.0	0.5	1.0	0.3	2.4	1.0	1.9	0.5	0.4	0.2	4.7	1.0
Profile recovery	49.1	130.1	65.9	145.0	37.1	96.3	53.7	113.3	31.6	156.8	66.7	228.4	48.6
Lower (7-30 cm) profile recovery	7.2	48.4*	24.7	15.0	3.8	32.0*	17.8	23.0	6.4	11.3	4.8	36.9*	7.8

[1] calculated on a 0.5 x 0.5 m plot size basis.

Asterisks indicate mean values significantly different from that of reference plot for each soil depth interval, t test at $p \leq 0.05$.

Table S3. Mean native soil organic carbon (non-biochar C, g) 4.5 years after biochar addition[1].

Soil depth (cm)	Reference	<0.5 mm BC		0.5–1 mm BC		1–5 mm BC	
	0% BC0	2% BC2	4% BC4	1.5% BC1.5	3% BC3	2% BC2	4% BC4
0 – 7	115.4	100.8	101.3	86.1*	96.4	98.8	83.2*
7 – 8	15.3	14.4	14.7	12.5*	13.4*	13.6	10.7*
8 – 9	14.3	13.3	15.6	11.9*	15.6	12.5	12.0*
9 – 10	16.0	13.4	15.1	11.4*	12.9*	12.3*	11.3*
10 – 11	15.1	13.2	16.5	13.2	13.1	12.2	11.8
11 – 12	16.1	13.2*	16.1	12.2*	13.4*	12.4*	13.0*
12 – 17	76.7	57.7*	65.7	58.8*	57.5*	53.8*	57.6*
17 – 25	98.0	81.8	111.1	82.4	73.6*	78.5*	80.8
25 – 30	59.4	53.3	58.1	40.1	36.2*	36.1*	41.6
Total	426.3	356.7*	414.2	328.6*	332.3*	330.3*	322.0*

[1] calculated on a 0.5 x 0.5 m plot size basis.

Asterisks indicate mean values significantly different than that of reference plot for each soil depth interval, t test at $p \leq 0.05$.

Table S4. Comparison of mean biochar carbon (BC) recovery from soil profiles, 1 and 4.5 years after biochar application.

BC recovery after one year[1]					BC recovery after 4.5 years				
Soil depth (cm)	BC4 <0.5 mm rice husk BC (g[2])	% BC recovery	BC4 0.5–1 mm maize cob BC (g)	% BC recovery	Soil depth (cm)	BC4 <0.5 mm maize cob BC (g)	% BC recovery	BC3 0.5–1 mm maize cob BC (g)	% BC recovery
0 – 5	128.4	52.3	120.0	35.7	0 – 7	130.0	33.3	90.3	25.2
5 – 6	24.7	10.1	17.5	5.2	7 – 8	3.8	1.0	2.8	0.8
6 – 7	11.9	4.8	7.4	2.2	8 – 9	2.1	0.5	1.9	0.5
7 – 8	5.3	2.1	1.3	0.4	9 – 10	1.0	0.3	1.2	0.3
8 – 9	1.7	0.7	0.8	0.2	10 – 11	0.6	0.2	0.6	0.2
9 – 10	0.8	0.3	0.7	0.2	11 – 12	0.4	0.1	0.3	0.1
10 – 15	1.0	0.4	2.9	0.8	12 – 17	3.7	0.9	5.4	1.5
15 – 20	0.5	0.2	2.3	0.7	17 – 25	2.4	0.6	8.9	2.5
-	-	-	-	-	25 – 30	1.0	0.3	1.9	0.5
Total	174.3	71.4	152.9	45.5	Total	145.0	37.1	113.3	31.6

[1] Data obtained from Obia et al. (2017a) where biochar application depth was 0–5 cm instead of 0–7 cm in the current study. Coarse biochar (particle size 1–5 mm) is excluded from the comparison as this size fraction was not part of the one-year study. Also, only the comparable doses of 3–4% are included.

[2] calculated on a 0.5 x 0.5 m plot size basis.

Table S5. Compilation of data used in this study.

Depth	Bulk Density	$\delta^{13}C$	BC recovery (g^{1})	non BC recovery (g^{1})
Reference BC0				
0-7 cm	1.26	-18.25	23.1	115.8
0-7 cm	1.26	-16.99	56.8	113.0
0-7 cm	1.26	-17.40	45.7	117.5
7-8 cm	1.29	-19.14	0.7	15.4
7-8 cm	1.29	-17.84	4.4	15.3
7-8 cm	1.29	-19.37	0.3	15.2
8-9 cm	1.29	-18.92	1.2	14.6
8-9 cm	1.29	-18.46	2.4	14.7
8-9 cm	1.29	-18.81	1.3	13.5
9-10 cm	1.29	-19.50	0.0	16.4
9-10 cm	1.29	-19.97	0.0	16.4
9-10 cm	1.29	-19.16	0.7	15.1
10-11 cm	1.29	-19.57	0.0	17.1
10-11 cm	1.29	-18.42	2.3	14.1
10-11 cm	1.29	-19.43	0.1	14.1
11-12 cm	1.29	-20.57	0.0	17.4
11-12 cm	1.29	-19.34	0.3	15.8
11-12 cm	1.29	-19.72	0.0	15.2
12-17 cm	1.29	-21.71	0.0	91.9
12-17 cm	1.29	-19.12	3.7	68.9
12-17 cm	1.29	-19.58	0.0	69.3
17-25 cm	1.29	-19.49	0.1	92.8
17-25 cm	1.29	-19.68	0.0	105.8
17-25 cm	1.29	-20.20	0.0	95.5
25-30 cm	1.29	-18.91	3.3	38.6
25-30 cm	1.29	-19.39	0.7	50.9
25-30 cm	1.29	-22.52	0.0	88.7
Fine BC2				
0-7 cm	1.21	-16.36	79.8	110.8
0-7 cm	1.21	-16.15	77.4	96.2
0-7 cm	1.21	-15.98	84.6	95.4
7-8 cm	1.29	-16.50	9.2	13.7
7-8 cm	1.29	-15.53	15.2	13.5
7-8 cm	1.29	-16.67	9.6	15.9
8-9 cm	1.29	-17.75	3.4	11.4
8-9 cm	1.29	-17.37	5.4	13.6
8-9 cm	1.29	-17.67	4.8	14.9
9-10 cm	1.29	-17.70	3.8	12.0
9-10 cm	1.29	-17.95	*NA*	*NA*
9-10 cm	1.29	-18.39	2.6	14.8
10-11 cm	1.29	-18.26	2.6	13.2
10-11 cm	1.29	-17.88	4.1	14.9
10-11 cm	1.29	-18.23	2.4	11.5
11-12 cm	1.29	-18.19	2.6	12.2

11-12 cm	1.29	-18.41	2.3	13.5
11-12 cm	1.29	-17.87	3.8	13.9
12-17 cm	1.29	-18.30	12.4	65.0
12-17 cm	1.29	-18.18	10.8	50.5
12-17 cm	1.29	-18.24	11.6	57.7
17-25 cm	1.29	-18.71	9.5	80.8
17-25 cm	1.29	-18.72	9.7	83.2
17-25 cm	1.29	-18.23	16.5	81.5
25-30 cm	1.29	-20.00	0.0	61.3
25-30 cm	1.29	-20.30	0.0	59.7
25-30 cm	1.29	-18.99	2.8	39.1
Fine BC4				
0-7 cm	1.17	-15.12	134	95.3
0-7 cm	1.17	-15.34	117	93.9
0-7 cm	1.17	-15.39	139.1	114.8
7-8 cm	1.29	-18.11	3.1	13.7
7-8 cm	1.29	-17.63	5.3	16.0
7-8 cm	1.29	-18.22	2.9	14.5
8-9 cm	1.29	-18.85	1.6	16.8
8-9 cm	1.29	-18.33	2.9	15.8
8-9 cm	1.29	-18.64	1.8	14.3
9-10 cm	1.29	-18.76	1.6	14.8
9-10 cm	1.29	-19.09	0.8	15.3
9-10 cm	1.29	-19.27	0.5	15.3
10-11 cm	1.29	-19.21	0.7	16.1
10-11 cm	1.29	-19.47	0.1	19.6
10-11 cm	1.29	-18.93	1.1	13.7
11-12 cm	1.29	-19.02	1.1	15.7
11-12 cm	1.29	-19.60	0.0	15.5
11-12 cm	1.29	-20.04	0.0	17.1
12-17 cm	1.29	-19.32	1.8	69.2
12-17 cm	1.29	-19.29	2.0	69.0
12-17 cm	1.29	-18.67	7.3	58.8
17-25 cm	1.29	-20.66	0.0	126.4
17-25 cm	1.29	-20.11	0.0	121.3
17-25 cm	1.29	-18.91	7.3	85.6
25-30 cm	1.29	-20.44	0.0	62.9
25-30 cm	1.29	-19.62	0.0	62.9
25-30 cm	1.29	-19.06	3.0	48.6
Medium BC1.5				
0-7 cm	1.21	-16.07	64.9	77.0
0-7 cm	1.21	-16.16	63.1	78.8
0-7 cm	1.21	-16.59	64.9	102.4
7-8 cm	1.29	-18.07	2.5	11.0
7-8 cm	1.29	-17.39	4.9	12.8
7-8 cm	1.29	-17.13	6.4	13.9
8-9 cm	1.29	-17.67	3.3	10.2
8-9 cm	1.29	-17.65	3.8	11.4

8-9 cm	1.29	-18.32	2.7	14.1
9-10 cm	1.29	-17.52	3.1	8.8
9-10 cm	1.29	-17.68	3.5	11.0
9-10 cm	1.29	-18.78	1.5	14.3
10-11 cm	1.29	-17.61	3.4	10.1
10-11 cm	1.29	-19.01	1.0	15.1
10-11 cm	1.29	-19.51	0.0	14.5
11-12 cm	1.29	-17.73	2.9	9.4
11-12 cm	1.29	-19.04	0.9	13.6
11-12 cm	1.29	-18.75	1.6	13.6
12-17 cm	1.29	-18.04	12.8	53.3
12-17 cm	1.29	-18.96	5.2	67.4
12-17 cm	1.29	-18.84	5.4	55.9
17-25 cm	1.29	-18.74	10.0	88.0
17-25 cm	1.29	-19.20	3.6	86.7
17-25 cm	1.29	-18.58	10.2	72.4
25-30 cm	1.29	-18.06	7.2	29.9
25-30 cm	1.29	-19.68	0.0	48.4
25-30 cm	1.29	-19.72	0.0	41.9
Medium BC3				
0-7 cm	1.16	-15.79	93.4	95.4
0-7 cm	1.16	-15.77	99.9	101.1
0-7 cm	1.16	-16.08	77.7	92.8
7-8 cm	1.29	-18.97	1.1	14.1
7-8 cm	1.29	-17.22	5.8	13.2
7-8 cm	1.29	-18.65	1.6	12.9
8-9 cm	1.29	-19.30	0.4	14.8
8-9 cm	1.29	-18.03	3.9	16.1
8-9 cm	1.29	-18.96	1.2	15.9
9-10 cm	1.29	-18.78	1.4	12.5
9-10 cm	1.29	-18.91	1.1	13.1
9-10 cm	1.29	-18.95	1.0	13.2
10-11 cm	1.29	-18.90	1.0	11.9
10-11 cm	1.29	-19.92	0.0	15.8
10-11 cm	1.29	-19.02	0.8	11.8
11-12 cm	1.29	-20.30	0.0	15.2
11-12 cm	1.29	-19.03	0.8	11.8
11-12 cm	1.29	-19.58	0.0	13.2
12-17 cm	1.29	-20.01	0.0	62.9
12-17 cm	1.29	-18.37	9.0	50.7
12-17 cm	1.29	-18.69	7.2	58.9
17-25 cm	1.29	-18.53	10.4	69.6
17-25 cm	1.29	-18.99	5.9	81.8
17-25 cm	1.29	-18.52	10.5	69.5
25-30 cm	1.29	-19.90	0.0	41.9
25-30 cm	1.29	-18.89	2.9	32.6
25-30 cm	1.29	-18.92	2.9	34.2
Coarse BC2				

0-7 cm	1.21	-15.62	99.8	92.9
0-7 cm	1.21	-15.47	103.7	89.0
0-7 cm	1.21	-14.47	232.9	114.4
7-8 cm	1.29	-18.20	2.7	13.1
7-8 cm	1.29	-17.01	6.2	12.5
7-8 cm	1.29	-18.03	3.7	15.3
8-9 cm	1.29	-19.05	0.8	11.8
8-9 cm	1.29	-18.58	1.7	12.5
8-9 cm	1.29	-18.55	2.0	13.2
9-10 cm	1.29	-19.23	0.4	11.5
9-10 cm	1.29	-19.14	0.7	13.8
9-10 cm	1.29	-19.41	0.1	11.5
10-11 cm	1.29	-19.10	0.6	10.7
10-11 cm	1.29	-18.93	1.1	13.1
10-11 cm	1.29	-20.02	0.0	12.9
11-12 cm	1.29	-19.92	0.0	12.9
11-12 cm	1.29	-19.26	0.4	12.5
11-12 cm	1.29	-19.65	0.0	11.9
12-17 cm	1.29	-19.24	1.8	51.4
12-17 cm	1.29	-19.32	1.3	55.1
12-17 cm	1.29	-18.92	4.7	55.0
17-25 cm	1.29	-19.17	3.2	69.0
17-25 cm	1.29	-19.39	1.3	81.3
17-25 cm	1.29	-20.23	0.0	85.1
25-30 cm	1.29	-19.46	0.2	40.1
25-30 cm	1.29	-19.28	1.0	34.5
25-30 cm	1.29	-20.14	0.0	33.9
		Coarse BC4		
0-7 cm	1.16	-13.86	282.5	93.1
0-7 cm	1.16	-15.33	113.9	91.1
0-7 cm	1.16	-14.01	178.3	65.3
7-8 cm	1.29	-17.95	2.7	10.5
7-8 cm	1.29	-17.13	5.3	11.5
7-8 cm	1.29	-16.10	8.5	10.2
8-9 cm	1.29	-18.26	2.3	11.6
8-9 cm	1.29	-16.98	5.9	11.8
8-9 cm	1.29	-18.08	2.9	12.6
9-10 cm	1.29	-18.67	1.4	11.2
9-10 cm	1.29	-18.27	2.3	11.6
9-10 cm	1.29	-18.30	2.1	11.1
10-11 cm	1.29	-18.79	1.4	12.8
10-11 cm	1.29	-19.27	0.4	11.9
10-11 cm	1.29	-18.61	1.5	10.8
11-12 cm	1.29	-19.63	0.0	13.9
11-12 cm	1.29	-18.86	1.0	10.9
11-12 cm	1.29	-19.50	0.0	14.2
12-17 cm	1.29	-19.13	3.3	64.4
12-17 cm	1.29	-18.93	4.8	58.1
12-17 cm	1.29	-18.33	9.4	50.3

17-25 cm	1.29	-19.02	5.6	82.1
17-25 cm	1.29	-18.60	12.4	90.8
17-25 cm	1.29	-17.61	23.5	69.4
25-30 cm	1.29	-19.37	0.7	39.6
25-30 cm	1.29	-18.69	4.4	35.9
25-30 cm	1.29	-18.34	9.0	49.1
Plots Outside of Experimental Block				
Lateral 0.5m North	1.26	-20.68	0.0	125.7
Lateral 0.5m South	1.26	-18.78	11.0	103.7
Lateral 1m North	1.26	-21.00	0.0	123.5
Lateral 1m South	1.26	-19.56	0.0	101.4
Lateral 2m North	1.26	-19.10	7.0	125.3
Lateral 2m South	1.26	-19.90	0.0	123.5
Lateral 3m North	1.26	-20.81	0.0	125.7
Lateral 3m South	1.26	-20.90	0.0	105.8
Lateral 5m North	1.26	-21.27	0.0	121.3
Lateral 5m South	1.26	-21.55	0.0	112.5

1. Calculated on a 0.5 x 0.5 m plot size basis.
